# The High-Spin Heme *b*_L_ Mutant Exposes Dominant Reaction Leading to the Formation of the Semiquinone Spin-Coupled to the [2Fe-2S]^+^ Cluster at the Q_o_ Site of *Rhodobacter capsulatus* Cytochrome *bc*_1_

**DOI:** 10.3389/fchem.2021.658877

**Published:** 2021-05-07

**Authors:** Marcin Sarewicz, Sebastian Pintscher, Łukasz Bujnowicz, Małgorzata Wolska, Artur Osyczka

**Affiliations:** Department of Molecular Biophysics, Faculty of Biochemistry, Biophysics and Biotechnology, Jagiellonian University, Kraków, Poland

**Keywords:** cytochrome bc_1_ (complex III), electron transfer, quinol oxidation, semiquinone, electron paramagnetic resonance

## Abstract

Cytochrome *bc*_1_ (mitochondrial complex III) catalyzes electron transfer from quinols to cytochrome *c* and couples this reaction with proton translocation across lipid membrane; thus, it contributes to the generation of protonmotive force used for the synthesis of ATP. The energetic efficiency of the enzyme relies on a bifurcation reaction taking place at the Q_o_ site which upon oxidation of ubiquinol directs one electron to the Rieske 2Fe2S cluster and the other to heme *b*_L_. The molecular mechanism of this reaction remains unclear. A semiquinone spin-coupled to the reduced 2Fe2S cluster (SQ_o_-2Fe2S) was identified as a state associated with the operation of the Q_o_ site. To get insights into the mechanism of the formation of this state, we first constructed a mutant in which one of the histidine ligands of the iron ion of heme *b*_L_ *Rhodobacter capsulatus* cytochrome *bc*_1_ was replaced by asparagine (H198N). This converted the low-spin, low-potential heme into the high-spin, high-potential species which is unable to support enzymatic turnover. We performed a comparative analysis of redox titrations of antimycin-supplemented bacterial photosynthetic membranes containing native enzyme and the mutant. The titrations revealed that H198N failed to generate detectable amounts of SQ_o_-2Fe2S under neither equilibrium (in dark) nor nonequilibrium (in light), whereas the native enzyme generated clearly detectable SQ_o_-2Fe2S in light. This provided further support for the mechanism in which the back electron transfer from heme *b*_L_ to a ubiquinone bound at the Q_o_ site is mainly responsible for the formation of semiquinone trapped in the SQ_o_-2Fe2S state in *R. capusulatus* cytochrome *bc*_1_.

## Introduction

The cytochrome *bc*_1_ complex (Cyt*bc*_1_) is one of the enzymes involved in energy conversion that takes place in mitochondria and many prokaryotic respiratory chains and anoxygenic photosynthesis (Berry et al., 2000; Crofts, 2004; Sarewicz and Osyczka, 2015). Structurally and functionally very similar to Cyt*bc*_1_—cytochrome *b*_6_ *f* is a central enzyme of oxygenic photosynthesis in cyanobacteria and plants (Darrouzet et al., 2004; Baniulis et al., 2008; Hasan et al., 2013; Cramer and Hasan, 2016). Cyt*bc*_1_ catalyzes electron transfer from membrane-soluble electron carriers–quinone (Q) derivatives (usually ubiquinones) (Al-Attar and de Vries, 2013) to water-soluble electron carrier: cytochrome *c* (Bertini et al., 2006). The energy released during enzymatic reduction of cytochrome *c* by ubiquinol (UQH_2_) is used to transfer protons across the membrane, contributing to building up the protonmotive force (Hunte et al., 2003). Cyt*bc*_1_ is not operating as a typical proton pump that uses special proton channels but it utilizes quinone molecules to transport protons across the lipid bilayers (Al-Attar and de Vries, 2013). This transport is carried out by coupling two opposite redox reactions of quinones at the two catalytic sites that are located within the enzyme structure at the opposite sides of the membrane (Mitchell, 1975; Crofts et al., 1983). The UQH_2_ oxidation site (Q_o_ site) is located close to the *p* side of the membrane (the positively charged side), while the UQ-reduction site (Q_i_ site) is located close to the *n* side of the membrane (the negatively charged side) (Xia et al., 1997; Crofts and Berry, 1998). The Q_o_ and Q_i_ sites are electronically connected by two low-potential, low-spin hemes *b*: heme *b*_L_ [*E*_m,7_ ∼ −120 mV in *R. capsulatus* (Zhang et al., 2008)], which is adjacent to the Q_o_ site and heme *b*_H_ (*E*_m,7_ ∼ + 60 mV), which is adjacent to the Q_i_ site. The oxidation of UQH_2_ at the Q_o_ site is accompanied by the release of two protons to the bulk water at the *p* side. Oppositely, the reduction taking place at the Q_i_ site is associated with uptake of two protons from the *n* side of the membrane. This way the protons are transported through the membrane along with pairs of diffusing UQH_2_/UQ molecules.

The reduction of UQ at the Q_i_ site is a sequential process, involving two consecutive electron transfers from the same cofactor (heme *b*_H_). A stable semiquinone is an intermediate of this reaction (Robertson et al., 1984; Jünemann et al., 1998; Dikanov et al., 2004) and, as implicated from recent studies, a mechanism of its stabilization might involve polarization of charges within the ring (Pintscher et al., 2020). On the other hand, the oxidation of UQH_2_ at the Q_o_ site directs two electrons derived from the UQH_2_ into two separate cofactor chains. In this so-called electron bifurcation reaction one electron is transferred on the Rieske [2Fe-2S] cluster (2Fe2S) and further, through cytochrome *c*_1_ onto a water-soluble cytochrome *c* (or *c*_2_ in some bacteria, such as the *Rhodobacter* strains). The second electron is transferred to heme *b*_L_ and subsequently through heme *b*_H_ it reaches the Q_i_ site where UQ is reduced first to semiquinone form (USQ_i_) and then to UQH_2_.

Despite a long history of studying the mechanism of electron bifurcation, the involvement of a semiquinone intermediate (USQ_o_) in this reaction is still unclear and thus a matter of intense discussion (Osyczka et al., 2004; Osyczka et al., 2005; Cape et al., 2007; Zhang et al., 2007; Zhu et al., 2007; Crofts et al., 2013; Sarewicz et al., 2013; Crofts et al., 2017; Sarewicz et al., 2021). A general agreement has been reached that a superoxide generation by Cyt*bc*_1_ results from a reaction of USQ_o_ with molecular oxygen (Boveris and Cadenas, 1975; Ksenzenko et al., 1983; Muller et al., 2002; Borek et al., 2008; Dröse and Brandt, 2008; Sarewicz et al., 2010; Pagacz et al., 2021). However, trapping the intermediate USQ_o_ radical during the UQH_2_ oxidation and its detection by electron paramagnetic resonance (EPR) has been proven difficult and obtained results have often been disputable or contradictory (de Vries et al., 1981; Jünemann et al., 1998; Cape et al., 2006; Cape et al., 2007; Zhang et al., 2007; Zhu et al., 2007; Sarewicz et al., 2013; Vennam et al., 2013; Victoria et al., 2013; Pietras et al., 2016; Crofts et al., 2017; Sarewicz et al., 2017; Sarewicz et al., 2018; Bujnowicz et al., 2019). Additionally, the inability to generate USQ_o_ in the equilibrium redox titrations (Takamiya and Dutton, 1979; Sarewicz et al., 2018) implicated a concept of a high instability of the semiquinone and its reactivity was proposed to be a reason for superoxide production by Cyt*bc*_1_.

Detection of a small amount of USQ_o_ under nonequilibrium conditions in isolated, antimycin-inhibited Cyt*bc*_1_ has been reported in a few studies (Cape et al., 2007; Zhang et al., 2007; Vennam et al., 2013; Victoria et al., 2013). The presence of USQ_o_ in Cyt*bc*_1_ samples was assigned to a stigmatellin-sensitive, X-band EPR signal which is typical of an organic radical with a nearly isotropic *g* value ∼2.0 (Cape et al., 2007; Vennam et al., 2013). Although experimental evidences were not presented, generation of this signal was assumed to be associated with the oxidation of UQH_2_ by the 2Fe2S cluster under conditions in which the resulting USQ_o_ was unable to donate electron to heme *b*_L_ as this heme, in antimycin-inhibited enzyme, was expected to be in the reduced state after two consecutive electron transfers from the Q_o_ site (Cape et al., 2007; Crofts et al., 2017).

Our previous work reported an unusual EPR signal with an average *g* value less than 2, which originated from the Q_o_ site of the antimycin-inhibited Cyt*bc*_1_, isolated from *Rhodobacter capsulatus* (Sarewicz et al., 2013; Sarewicz et al., 2017; Bujnowicz et al., 2019)*.* This signal was detected only when the Q_o_ site was able to catalyze the UQH_2_ oxidation and the cytochrome *c* reduction before the system reached equilibrium. The signal was not present in the samples containing specific inhibitors of the Q_o_ site (stigmatellin, myxothiazol, famoxadone, azoxystrobin, kresoxim-methyl, etc.), nor in the mutants with disabled Q_o_ site (like, for example, *cytb*:G158W). Simulations of the EPR spectra suggested that this signal results from the spin-spin exchange interactions between USQ_o_ and the reduced 2Fe2S (it is thus referred to as SQ_o_-2Fe2S) with approximated *J* constant ∼3.6 GHz (Sarewicz et al., 2013). This suggestion was further supported by the observations that the *g* values of the SQ_o_-2Fe2S state were frequency-dependent—they changed upon increasing the microwave frequency from X-band ∼9.4 to Q-band ∼33.5 GHz. At X-band, the most prominent “derivative-shaped” transition of SQ_o_-2Fe2S in Cyt*bc*_1_ was detected at *g* = ∼1.94 and was found to shift to ∼1.96 at Q-band. Remarkably, the SQ_o_-2Fe2S signal was also detected in isolated but non-inhibited spinach cytochrome *b*_6_ *f* during the oxidation of synthetic decylplastoquinol and the reduction of plastocyanin (Sarewicz et al., 2017).

Our subsequent work revealed that the SQ_o_-2Fe2S signal can be generated in antimycin-supplemented native chromatophore membranes isolated from *R. capsulatus,* in which the reactions at the Q_o_ site are triggered by the photosynthetic reaction center (RC) (Sarewicz et al., 2018). In these experiments, the characteristic transitions of the SQ_o_-2Fe2S state with a readily detectable maximum in continuous wave X-band spectra at g = 1.95 were detected during the redox-titration of chromatophores in relatively narrow ranges of the external redox potential (*E*_h_). However, the signal appears only when the chromatophores are illuminated by continuous light during the titrations, which keeps the RC activated to supply substrates for the Q_o_ site. Similar titrations performed in dark, i.e. without activation of RC (equilibrium conditions), do not lead to the generation of SQ_o_-2Fe2S. Clearly, in membranes, similarly to isolated Cyt*bc*_1_, the SQ_o_-2Fe2S state corresponds to one of the states of the enzyme that can be populated to detectable levels only under nonequilibrium conditions.

One of the most important observations regarding the enzymatic state of *Rhodobacter capsulatus* Cyt*bc*_1_ is that the SQ_o_-2Fe2S state is detected in samples in which heme *b*_L_ remains oxidized (Sarewicz et al., 2013; Sarewicz et al., 2018). This suggests that in those experiments, the reaction sequence leading to the formation of the SQ_o_-2Fe2S state begins with the electron transfer from the reduced heme *b*_L_ to the UQ molecule bound at the Q_o_ site. The resulting SQ_o_ then interacts with the reduced 2Fe2S that is present at the Q_o_ site. However, a direct evidence supporting this mechanism has not yet been provided. Furthermore, the SQ_o_ can, in principle, also be formed in another reaction: electron transfer from UQH_2_ to oxidized 2Fe2S, which would also result in the formation of the SQ_o_-2Fe2S state if the subsequent electron transfer from SQ_o_ to heme *b*_L_ is (somehow) blocked. In this work, we addressed this issue with a series of experiments performed with the histidine ligand mutant of *R. capsulatus* in which the heme *b*_L_ was inactivated. The mutation of choice was patterned after the previous study with closely related *Rhodobacter sphaeroides* where replacing the histidine ligand with asparagine (*cytb*:H198N) was found to result in a loss of spectral properties of heme *b*_L_ typical of WT Cyt*bc*_1_ and in a complete loss of the enzymatic activity (Yang et al., 2008). We found that in *R. capsulatus* the H198N mutation converted the low-spin heme *b*_L_ into the high-spin heme unable to transfer electron from SQ_o_ to heme *b*_H_, and such conditions were tested for the efficiency of SQ_o_-2Fe2S generation in chromatophores in dark or illuminated by light. The tests failed to detect the spin-spin–coupled SQ_o_-2Fe2S state in H198N even in chromatophores activated by light, which we considered an indication that electron transfer from heme *b*_L_ to the UQ molecule at the Q_o_ site is mainly responsible for creating conditions that ultimately lead to the formation of *R. capsulatus.*

## Materials and Methods

### Construction of the H198N Mutant

The H198N mutant of *Rhodobacter capsulatus* containing the H198N mutation in cytochrome *b* subunit was generated using the genetic system described previously (Atta-Asafo-Adjei and Daldal, 1991). Mutation H198N was constructed by the QuikChange site-directed mutagenesis kit from Stratagene using pPET1-ST (Czapla et al., 2012b) as the template, and the mutagenic forward H198N-F: 5′-TTC TTC TCG CTG **A**AC TAT CTG CTG CCC TTC G -3′ and reverse H198N-R 5′-GGG CAG CAG ATA GT**T** CAG CGA GAA GAA GCG G -3′ oligonucleotides. After sequencing, the *Xma*I/*Sfu*I fragment of pPET1-ST-H198N plasmid bearing the desired mutation and no other mutations was exchanged with its wild-type counterparts in pMTS1. This created the expression vector pMTS1-ST-H198N that was introduced into *R. capsulatus* MT-RBC1 strain (*pet*ABC-operon deletion background) via triparental crosses (Atta-Asafo-Adjei and Daldal, 1991). The presence of H198N mutation was confirmed by sequencing the plasmid DNA isolated from the mutated *R. capsulatus* strain (H198N mutant).

### Isolation of Chromatophores

Chromatophores from *R. capsulatus* strains were prepared as described in detail by Sarewicz et al. (2018). Briefly, bacterial cultures were grown heterotrophically under semiaerobic conditions on the MPYE medium. After 48 h of growing the bacteria cells were centrifuged for 30 min at 6,641 g and the pellet resuspended in buffer containing 50 mM MOPS (pH 7), 100 mM KCl, and 1 mM EDTA, followed by the addition of protease inhibitors (benzamidine, PMSF, and 6-aminocaproic acid). The cells’ suspension was then passed two times through French Press® with pressure maintained between 11 and 15 MPa. Bacterial lysate was then centrifuged for 30 min at 24,104*g* and the obtained supernatant was then ultra-centrifuged at 244,062*g* for 1.5 h. The pellet containing isolated chromatophores was resuspended and homogenized in 50 mM bicine buffer (pH 8) containing 100 mM KCl and 1 mM EDTA.

### Redox Titrations

The prepared chromatophores membranes containing WT and mutant H198N Cyt*bc*_1_ were supplemented with antimycin at a final concentration of 0.15 mM and redox titrated under anaerobic conditions in dark. The solutions contained the following redox mediators: 50 µM 2,3,5,6-tetramethyl-1,4-phenylenediamine (DAD), 50 µM 1,2-naphthoquinone (NQ), 50 µM 1,2-naphthoquinone-4-sulfonate (NQS), 12.5 µM phenazine ethosulfate (PES), 25 µM phenazine methosulfate (PMS), 50 µM duroquinone (DQ), 12.5 µM indigotrisulfonate (ITS), 50 µM anthraquinone-2-sulfonic acid (AQS), 50 µM 2-hydroxy-1,4-naphthoquinone (HNQ), and 50 µM benzyl viologen (BV). The external redox potential (*E*_h_) of the buffer was adjusted by injection of small volumes of sodium dithionite or potassium ferricyanide solutions. In dark equilibrium redox titration for each *E*_h_ about ∼200 µL of the chromatophore solution was collected and injected to an argon-flushed EPR quartz tube and then subsequently frozen in cold isopentane. The illuminated samples were prepared by inserting the EPR tube containing chromatophores to a home-built cylindrical chamber blocked at the bottom with a piston connected to an electromagnet. The chamber contained 43 diodes (OSRICA3131A by Optosupply), having a single emission band at 850 nm, that were built in the walls of the chamber. The tubes were illuminated for 5 s and then were released to cold isopentane bath by pulling the piston of the electromagnet. A freezing time was estimated to be ∼ 1–2 s. With this method a maximum level of SQ_o_-2Fe2S is reached after 2 s of illumination, as determined for chromatophores containing antimycin-inhibited WT Cyt*bc*_1_. A decay time constant of the SQ_o_-2Fe2S state after switching off the light is about 1.8 s^−1^, which means that not more than ∼60% of the SQ_o_-2Fe2S state can be trapped after 1–2 s of the deadtime associated with the freezing of the samples.

We note that isolation of chromatophores required special care to prevent loss of cytochrome *c*_2_ during the cell centrifugations with an excessive RCF. This is because the formation of the SQ_o_-2Fe2S center in light-activated chromatophores was found to be sensitive to the coupling of RC with Cyt*bc*_1_ by cytochrome *c*_2_. This effect is illustrated in Supplementary Figure S1, which demonstrates a decrease or even complete disappearance of the SQ_o_-2Fe2S state in chromatophores having a shortage of cytochrome *c*_2_. A partial restoration of the signal is possible upon the addition of external horse cytochrome *c* to the chromatophores followed by the sonication of the samples.

### Preparation of Isolated WT and H198N Enzymes

Cyt*bc*_1_ complexes were isolated from detergent-solubilized chromatophores by Strep-tag^®^ affinity chromatography, using the procedure described by Czapla et al. (2012a). Purity of the isolated proteins was checked with SDS–PAGE (Supplementary Figure S2). Cyt*bc*_1_ samples were concentrated on centrifugal filter units and dialyzed against buffer composed of 50 mM Tris (pH 8), 100 mM NaCl, 1 mM EDTA, 20% glycerol (v/v), and 0.01% (m/m) DDM. The concentration of the isolated cytochrome *bc*_1_ was determined as described by Valkova-Valchanova et al. (1998).

#### EPR Measurements

All the measurements were performed using a Bruker ElexSys E580 spectrometer equipped with a super-HighQ (SQHE0511) resonator and ESR900 cryostat unit (Oxford Instruments). The chromatophore membranes were measured using the parameters described by Sarewicz et al. (2018). Briefly, the temperature of the measured samples was 20 K, microwave power of 2 mW and frequency 9.4 GHz, modulation amplitude of 14.36 G, sweep width of 926.8 G, sweep time of 40.96 s, and time constant of 81.92 ms. The number of scans was usually 1–3 depending on the signal-to-noise (S/N) ratio. The registered spectra were processed and analyzed using the Eleana EPR program.

The isolated WT and H198N Cyt*bc*_1_ complexes were measured as follows. The X-band EPR spectra of low- and high-field transitions of hemes were measured at 10 K, with 2 mW microwave power, frequency of 9.4 GHz, and modulation amplitude of 6 G. The X-band EPR spectra of the 2Fe2S cluster was measured at 30 K with 0.65 mW microwave power and modulation amplitude of 16 G.

## Results and Discussion

### H198N Mutant Incorporates a High-Potential, High-Spin Heme *b*_L_

**FIGURE 1:**
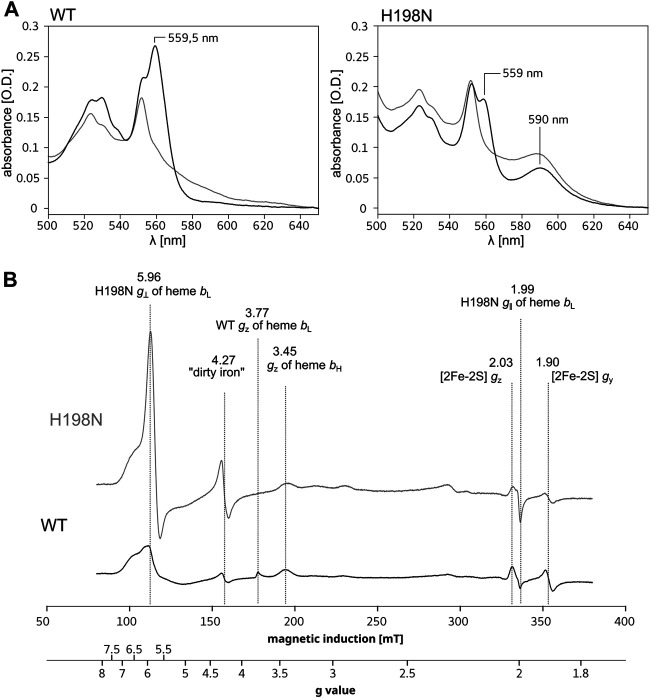
Comparison of spectra for isolated WT and H198N mutants of Cyt*bc*_1_. **(A)** Optical spectra of WT (left) and H198N mutant (right) measured for ascorbate (gray) and dithionite-reduced (black) Cyt*bc*_1_. Maximum at ∼560 nm originates from absorption of hemes *b*. Additional absorption band at ∼590 nm is likely to originate from heme *b*_L_ with changed ligand in H198N mutant. **(B)** X-band EPR spectra of air-oxidized WT (black) and H198N mutant (gray) measured at 10 K. In the range between ∼70 and ∼160 mT the contribution from high-spin iron centers is detected. In the range between ∼160 and 200 mT, the *g*_z_ transitions of HALS hemes *b* transitions are detected, while the reduced 2Fe2S contributes to transitions at the magnetic field above ∼320 mT.

Cyt*bc*_1_ contains the low-potential [*E*_m,7_ = ∼ −120 mV (Zhang et al., 2008)], low-spin (L-S) heme *b*_L_ which is axially coordinated by two histidine side chains: H97 and H198 (*R. capsulatus* numbering of cyt*b* subunit) (Berry et al., 2004). This heme is diamagnetic in the reduced state and, together with a higher potential (*E*_m_ = +60 mV) heme *b*_H_, contributes to the absorbance band with a maximum at ∼560 nm of the fully reduced WT Cyt*bc*_1_ (Figure 1A, left, black). A replacement of histidine 198 to non-liganding asparagine is expected to significantly alter properties of heme *b*_L_ or even prevent its incorporation into the protein, as previously reported for the ligand mutants of hemes *b* in *R. capsulatus* and *R. sphaeroides* (Yang et al., 2008). Indeed, the optical spectrum of the H198N mutant in *R. capsulatus* constructed in this work (^Rc^H198N) exhibits a significant decrease in the absorbance at ∼560 nm (Figure 1A, right, black), consistent with the previous observation on the analogical H198N mutant in *Rhodobacter sphaeroides* (^Rs^H198N) (Yang et al., 2008). Such a decrease suggests that one of the L-S hemes *b* might be missing, as was concluded for the ^Rs^H198N mutant. However, along with the disappearance of the absorption band at ∼560 nm we note an additional absorption maximum at ∼590 nm present in both the ascorbate- and dithionite-reduced samples containing ^Rc^H198N. This band was not seen in WT and suggested that it is associated with a change in the heme ligand in the ^Rc^H198N mutant.

Low-temperature X-band EPR spectra for isolated WT and the H198N mutant for air-oxidized samples are compared in Figure 1B. WT Cyt*bc*_1_ shows the *g*_z_ transitions typical of highly anisotropic low-spin (HALS) hemes at *g* = 3.77 and 3.45 (Figure 1B, black), originating from the oxidized, low-spin hemes *b*_L_ and *b*_H_, respectively (Salerno, 1984; Finnegan et al., 1996). From those two transitions, only the *g* = 3.45 originating from heme *b*_H_ was detected in H198N mutant (Figure 1B, gray), indicating that this heme was properly assembled into the protein core and retained its native bis–his axial ligation. The missing *g* = 3.77 transition was consistent with the expected lack of HALS heme *b*_L_ with his–his axial ligation. Instead, a relatively intense transition at *g* = ∼5.96 and a much weaker at *g* = ∼2 were detected revealing that in ^Rc^H198N, the HALS heme *b*_L_ was converted into an axial, high-spin (H-S) form. Given the fact that other centers contribute to the EPR signal in the region of *g* = 5.96, the heterogeneity, if any, of the spin state of heme *b*_L_ in the mutant cannot be evaluated.

To get insights into the redox properties of hemes *b* in the H198N mutant, we analyzed the relaxation properties of a semiquinone formed at the Q_i_ site, which, as reported recently, strongly depend on whether the electron preferentially stays on heme *b*_H_ or heme *b*_L_, thus can be used to estimate the relative difference in equilibrium redox potentials between these hemes (Pintscher et al., 2018). The results, discussed in detail in SI (Supplementary Figures S3 and S4), revealed that heme *b*_L_ in the mutant has a redox potential much more positive than the native HALS heme *b*_H_ (Pintscher et al., 2018). Furthermore, the *g* = 5.96 transition disappeared when the ^Rc^H198N Cyt*bc*_1_ was reduced by a synthetic substrate—decylubiquinol (Supplementary Figure S5), which suggests that heme *b*_L_ in ^Rc^H198N mutant has a much higher redox midpoint potential than heme *b*_L_ of the wild type.

### H198N Mutant Binds Properly UQ to the Q_o_ Site

**FIGURE 2:**
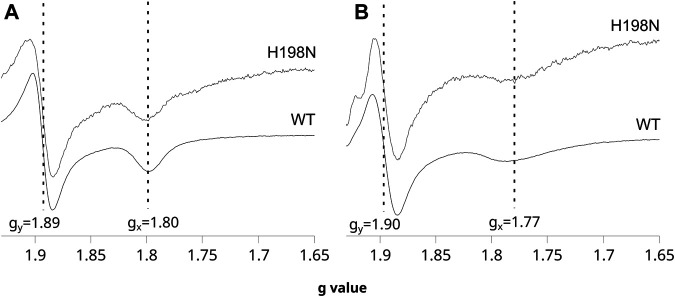
Sensitivity of the EPR spectra of the 2Fe2S cluster to changes in redox state of the Q pool for WT and the H198N mutant at pH 8. **(A)** The spectra measured for chromatophores poised at + 200 mV (Q pool oxidized) in dark. **(B)** The spectra measured for chromatophores poised at + 20 mV (Q pool half reduced). The vertical lines indicate the positions of *g*_y_ and *g*_x_ transitions of the 2Fe2S cluster, which shifts to higher and lower *g* values, respectively, upon the reduction of the Q pool. The spectra of the mutant were magnified ∼5 times to normalize the *g*_y_ signals to the same level as in WT.

The shape of the CW EPR spectra of the reduced 2Fe2S cluster in chromatophores is sensitive to the type of molecule that occupies the Q_o_ site. In particular, the relatively narrow *g*_x_ transition at 1.80 indicates that UQ is bound at this site and interacts with the cluster, whereas a more broad *g*_x_ = 1.77 indicates the presence of UQH_2_ in the Q_o_ site (Robertson et al., 1993; Ding et al., 1995; Cooley et al., 2004; Sharp et al., 1999). This change in *g*_x_ transition is so characteristic that it was often used in evaluating the effects of mutations on the binding of ubiquinone molecules to the catalytic site, or in the redox titrations of ubiquinones in the membranes (Cooley et al., 2004; Osyczka et al., 2006). As shown in Figure 2A, the CW EPR spectra of the ascorbate-reduced 2Fe2S cluster in chromatophores containing WT and H198N Cyt*bc*_1_ were similar and both exhibited clear *g*_x_ = 1.80 transition when the Q pool was fully oxidized (at high *E*_h_). Furthermore, reducing the Q pool, upon decreasing the external redox potential, changed the *g*_x_ from 1.80 to ∼1.77 in both WT and the mutant protein (Figure 2B). These observations indicate that UQ and UQH_2_ can bind to the Q_o_ site of the mutant in the same way as in WT [see references Cooley et al. (2004), Osyczka et al. (2006) for details]. This feature, along with the specifically altered redox properties of heme *b*_L_ described earlier, made H198N an attractive mutant for the mechanistic analysis of the formation of the SQ_o_-2Fe2S state.

### Controlling the Efficiency of SQ_o_-2Fe2S Generation in Illuminated Chromatophores

In our previous studies we established a general methodology for monitoring the formation of the SQ_o_-2Fe2S state in chromatophore membranes (Sarewicz et al., 2018). We found that this state can be detected by CW EPR during the redox titrations of chromatophores containing antimycin-inhibited Cyt*bc*_1_ only under nonequilibrium conditions when the photosynthetic reaction center and allied Cyt*bc*_1_ were kept activated by continuous light during the titration. The same experiments performed in dark (equilibrium conditions) do not lead to the formation of SQ_o_-2Fe2S at detectable levels (Sarewicz et al., 2018).

**FIGURE 3:**
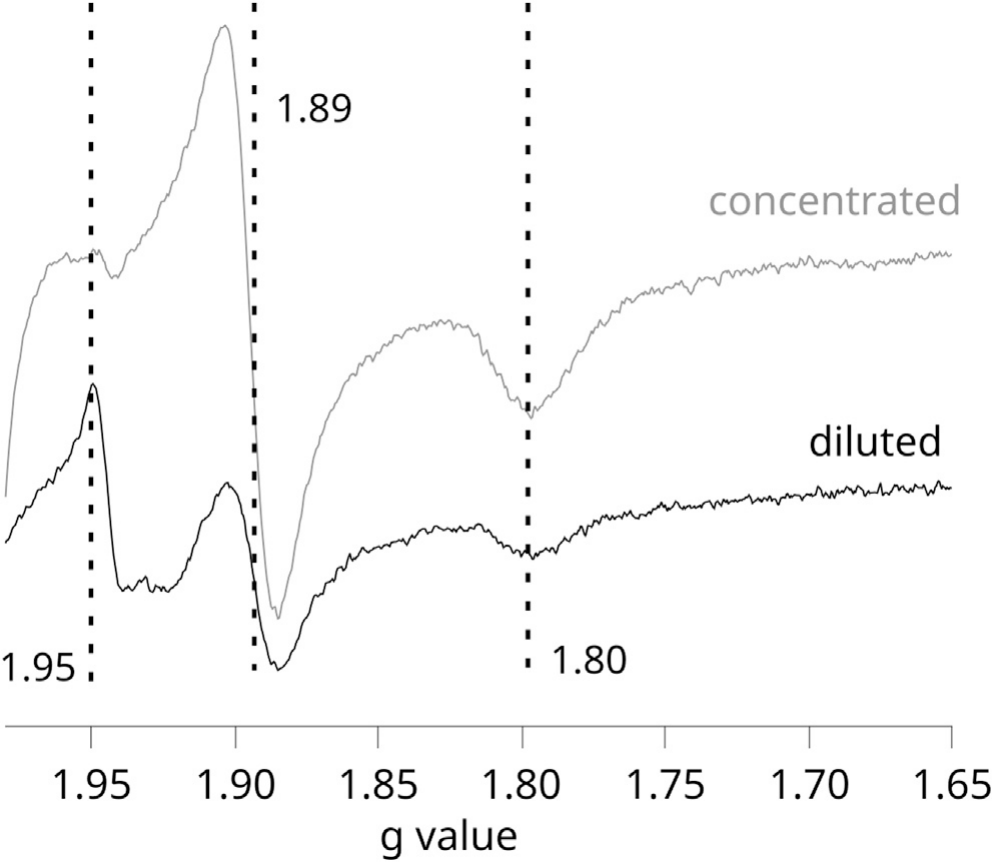
X-band EPR spectra measured for illuminated chromatophores containing WT Cyt*bc*_1_ at pH 8 and *E*_h_ = +77 mV. Gray and black spectra were obtained for samples containing 40 and 20 μM, respectively. Dashed vertical lines show *g*_y_ = 1.89 and *g*_x_ = 1.80 transitions of 2Fe2S cluster. The maximum at 1.95 originates from SQ_o_-2Fe2S.

Given that in these experiments, the formation of SQ_o_-2Fe2S strongly depends on the activation level of RC, any comparative redox titrations of chromatophores illuminated by continuous light (nonequilibrium conditions) require careful controls to assure that conditions for the generation of the SQ_o_-2Fe2S signal and its detection were equally met in each of the studied cases. We found that the most important factor influencing the efficiency of SQ_o_-2Fe2S generation in chromatophores was the optical transparency for the light that activates RC. This effect is shown in Figure 3, which compares the two EPR spectra obtained for the concentrated (gray) and diluted (black) samples of the same batch of chromatophores (containing 40 and 20 μM cytochrome *c*_1_, respectively).

This example emphasizes that the SQ_o_-2Fe2S signal (*g* = 1.95) can be lost in the more concentrated sample despite improvement in the signal-to-noise ratio, and this is related to the fact that the activation of RC is much weaker due to increased opacity of the solution. The weaker light activation of RC in the more concentrated samples was also reflected in the measured UQ/UQH_2_ average redox midpoint potential, which, despite illumination of the sample, approached those detected in dark. Thus, the titrations of the SQ_o_-2Fe2S signal on illuminated chromatophores required sufficiently diluted samples. However, after diluting the samples, a measurement of the redox state of hemes *b* by EPR is compromised due to the fact that the amplitudes of these hemes are relatively very weak. We found that at the concentrations of Cyt*bc*_1_ at which hemes *b* became clearly detectable by EPR, the SQ_o_-2Fe2S signal started to disappear due to increase of the opacity of the samples. Therefore, the experiments were limited to a relatively narrow range of Cyt*bc*_1_ concentrations that allowed efficient activation of RC and, at the same time, a detection of relatively good-quality EPR spectra.

### Light-Activated RC Interacts With H198N in Chromatophores and Imposes a Shift in Apparent Midpoint Potential of the 2Fe2S Cluster

In view of the observation that the efficiency of SQ_o_-2Fe2S generation in the light-activated WT chromatophores strongly depends on the optical transparency of the samples (see the paragraph above), it became apparent that prior to comparing WT and the H198N mutant, it was necessary to verify that RC in both WT and the H198N mutant containing chromatophores were sufficiently well activated by light and, consequently, both the systems were pushed out of equilibrium. Furthermore, it was necessary to apply a verification method that was independent of the analysis of the SQ_o_-2Fe2S signal. One such possibility relates to the fact that light activation affects the redox state of the membranous Q pool. Changes in the redox state of the Q pool can be monitored by measuring a progressive decrease in the amplitude of the normalized *g*_x_ = 1.80 transition of the 2Fe2S cluster as the Q pool becomes progressively reduced (Sarewicz et al., 2018). Illumination leads to the electron transfer from the cytochrome *c*_2_ pool to the Q pool and the extent of the transfer is reflected in a shift in the Nernst curve of UQ/UQH_2_ couple toward higher values. Thus, the shift confirms the activation level of RC.

**FIGURE 4:**
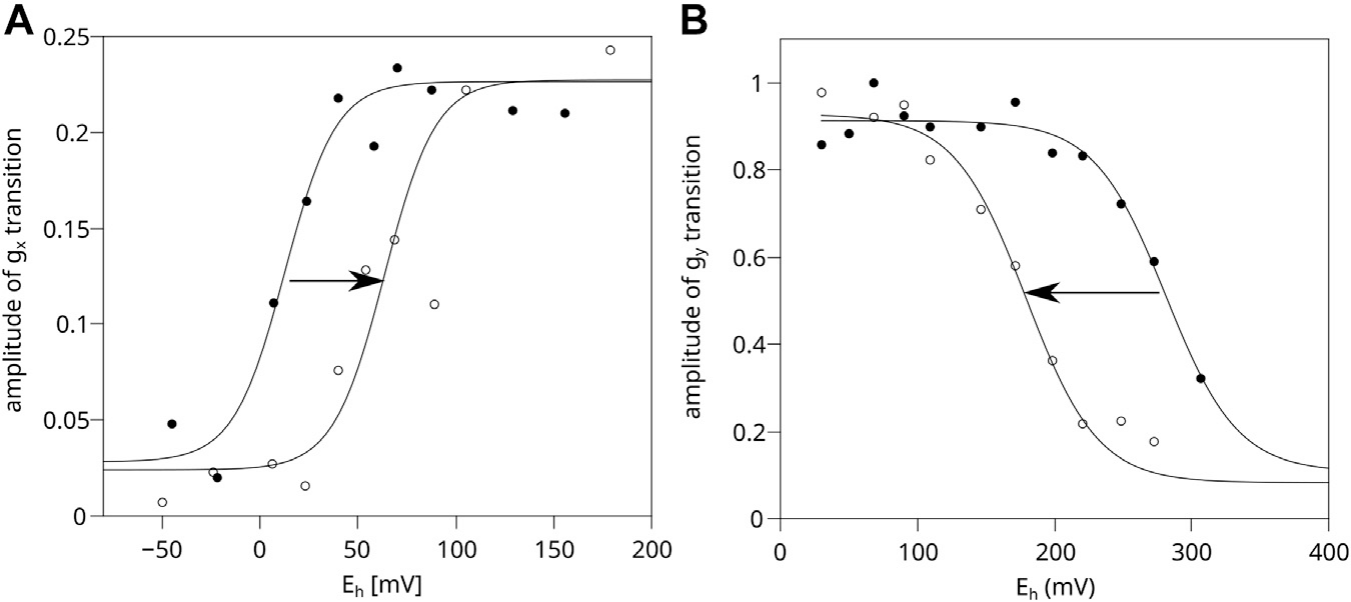
Comparison of efficiency of light activation of RC in chromatophores containing WT and H198N mutant of Cyt*bc*_1_. **(A)** Amplitude of the normalized *g*_x_ transition of the 2Fe2S cluster of WT Cyt*bc*_1_ which is proportional to the amount of UQ in the Q pool in dark (closed circles) and in illuminated samples (open circles). The experiential data points were simulated using an appropriate Nernst equation with *n* = 2. **(B)** Amplitude of the *g*_y_ transition of the 2Fe2S cluster measured for the illuminated chromatophores containing H198N mutant of Cyt*bc*_1_ (open circles) and chromatophores in dark (closed circles).

Figure 4A shows that in chromatophores containing WT Cyt*bc*_1_ at a concentration equal to 20 μM cytochrome *c*_1_ (corresponding to black spectrum of Figure 3), the obtained equilibrium redox potential of UQ/UQH_2_ couple in dark shifts by approximately +60 mV upon the activation of RC by light. Clearly, the optical transparency of the chromatophores in this case was enough to allow sufficient RC activation. In a more concentrated sample (corresponding to the gray spectrum of Figure 3) the measured UQ/UQH_2_ average redox midpoint potential was similar in light and in dark, and the lack of the shift (lack of activation of RC) was concomitant with the lack of the SQ_o_-2Fe2S signal (data not shown).

In the case of the H198N mutant, the expression level of Cyt*bc*_1_ was found to be lower by approximately 2–3 folds than WT (data not shown). Thus, after concentrating the H198N chromatophores to reach 20 μM of cytochrome *c*_1_, the samples became too opaque to allow efficient activation of RC. As a result, the illumination of the samples did not lead to a shift of the UQ/UQH_2_ Nernst curve toward a higher value (data not shown). Therefore, the chromatophores containing H198N must have been diluted to 10 μM of cytochrome *c*_1_ to reach the optical transparency similar to that of WT chromatophores. This, in turn, exerted a negative effect of the quality of the measured EPR spectra of 2Fe2S cluster, and the reliable measurement of *g*_x_ transition was not possible, precluding the possibility of monitoring activation level by determining the shift in the redox state of Q pool.

Instead, we monitored the extent of 2Fe2S oxidation upon activation of RC by comparing the amplitude of the relatively strong *g*_y_ transition of the 2Fe2S cluster, as a function of *E*_h_ for illuminated chromatophores with those measured in dark. Illumination of H198N chromatophores imposed a shift of the Nernst curve with the midpoint potential of the 2Fe2S cluster from approximately +300 mV (in dark) to approximately +180 mV (in light) (Figure 4B, dashed and solid lines, respectively). This shift was an expected effect if RC had been efficiently activated and able to oxidize the whole chain of high-potential cofactors connecting RC and Cyt*bc*_1_ (i.e., cytochrome *c*_2_/*c*_1_ and 2Fe2S). We note that similar shift in the 2Fe2S cluster can be observed in WT chromatophores; however, Cyt*bc*_1_ must be inhibited by myxothiazol (i.e., it is not observed in non-inhibited Cyt*bc*_1_). This inhibitor binds at the Q_o_ site and prevents oxidation of UQH_2_ at this site; however, it does not impair the movement of the 2Fe2S cluster nor its interaction with other components of the high-potential chains (Kim et al., 1998; Darrouzet et al., 2000; Esser et al., 2004; Osyczka et al., 2004; Sarewicz et al., 2009; Cieluch et al., 2010). Thus, the observed shift in H198N chromatophores not only confirms the activation of RC, but also indicates that the components of the high-potential chain in this mutant are functionally linked with RC. At the same time, these components remain decoupled from the low-potential chain of Cyt*bc*_1_ because of the enzymatic incompetence of mutated heme *b*_L_ (with the net effect similar to the presence of myxothiazol in the Q_o_ site of the native enzyme). We note that the observed shift in the 2Fe2S cluster induced by light activation of RC is interesting itself, and it will be discussed elsewhere.

### H198N Fails to Generate Detectable Amounts of SQ_o_-2Fe2S Spin-Coupled State

Having established the conditions for meaningful comparison of WT and H198N chromatophores (see the previous paragraphs), we performed a series of redox titrations that screened for the SQ_o_-2Fe2S state in the H198N mutant.

**FIGURE 5:**
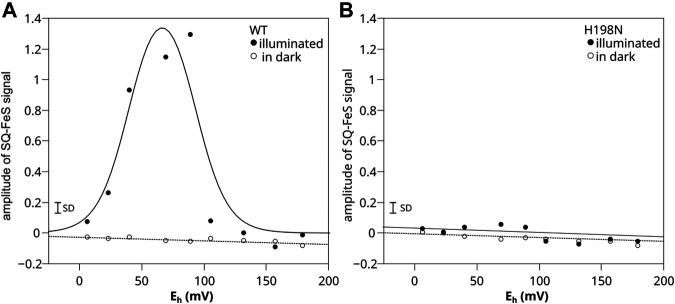
Comparison of SQ_o_-2Fe2S generation in chromatophores containing WT **(A)** and H198N mutant **(B)** of Cyt*bc*_1_. Amplitude of SQ_o_-2Fe2S was measured for illuminated samples (closed circles) and titrated in dark (open circles). Optical transparencies were the same for A and B, and the concentrations of cytochrome *c*_1_ were 20 and 10 μM, respectively. The solid line in A represents the fit of *f*_SQ_o_-2Fe2S_ = *f*_A_/{1 + exp [0.039*n* (*E*_h_ − *E*_1_)] + exp [0.039*n* (*E*_2_ − *E*_h_)]} function described in detail by Sarewicz et al. (2018). The fit yielded *E*_1_ = +92 ± 8 mV and *E*_2_ = +40 ± 7 mV for *n* = 2 and *f*_A_ = 1.7 ± 0.4. The small bar denoted marked as SD shows the typical uncertainty of the amplitude reads and includes the baseline and the noise of the EPR spectrum. It does not include uncertainty related to changes in efficiency of SQ_o_-2Fe2S generation dominated by variations in light activation and/or changes in the freezing time.

The results for WT chromatophores were consistent with the previous observations (Sarewicz et al., 2018) (Figure 5A). When titrations were performed in light at pH 8, the SQ_o_-2Fe2S was detected within the range of *E*_h_ below +150 mV and above 0 mV (with the maximum amplitude at *E*_h_ ∼ +66 mV) (closed circles). The SQ_o_-2Fe2S was not detected in this range of *E*_h_ when similar titrations were performed in dark (open circles).

Remarkably, in titrations performed with chromatophores containing H198N instead of WT Cyt*bc*_1_, the SQ_o_-2Fe2S was not detected at all, regardless of whether the experiment was conducted in light or in dark (Figure 5B). A corollary of this result on the mechanisms of the formation of the SQ_o_-2Fe2S state in antimycin-inhibited WT Cyt*bc*_1_ is discussed below.

### The H198N Mutation Implicates That Semireverse Mechanisms of SQ_o_-2Fe2S Formation Are Dominant in WT Cyt*bc*_1_

**FIGURE 6:**
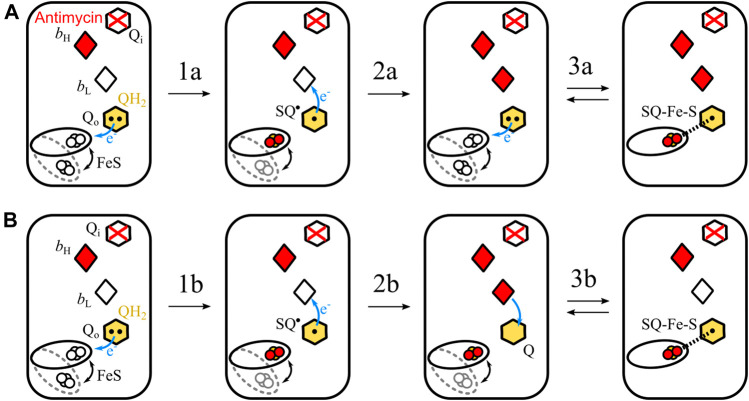
Simplified scheme of two possible reactions of SQ_o_-2Fe2S formation. **(A)** The *semiforward* mechanism of UQH_2_ oxidation by the 2Fe2S cluster at the time when heme *b*_L_ is unable to accept electron (because of being already reduced) from resulting SQ_o_, leading to the creation of the SQ_o_-2Fe2S state. **(B)** The *semireverse* mechanism assumes that the reduced heme *b*_L_ donates electrons to UQ at the time when the reduced 2Fe2S is close to the Q_o_ site. This leads to the creation of the SQ_o_-2Fe2S state. According to the mechanism shown in A, the redox-active heme *b*_L_ is not necessary, while the mechanism shown in B requires heme *b*_L_ for the generation of the SQ_o_-2Fe2S state.

Let us consider two possible mechanisms of the formation of SQ_o_-2Fe2S in antimycin-inhibited Cyt*bc*_1_ shown schematically in Figure 6. In each of these two scenarios it is assumed that heme *b*_H_ is already reduced after the oxidation of the first UQH_2_ molecule at the Q_o_ site. This heme cannot be reoxidized by electron transfer to the Q_i_ site, because this site is occupied by antimycin.

The first mechanism assumes the sequence of reactions schematically shown in Figure 6A. The UQH_2_ binds at the Q_o_ site where it undergoes one-electron oxidation by 2Fe2S forming an unstable semiquinone, SQ_o_ (transition 1a). This semiquinone immediately donates electron to heme *b*_L_ and the resulting UQ molecule is then replaced with next UQH_2_ (transition 2a in Figure 6A). As the Q_i_ site is blocked by antimycin, both hemes *b*_H_ and *b*_L_ cannot be reoxidized by electron transfer to the Q_i_ site. Thus, SQ_o_ formed upon the oxidation of the second UQH_2_ molecule by the 2Fe2S cluster is unable to donate electron to heme *b*_L_ and SQ_o_ and the reduced cluster forms spin-coupled state (transition 3a). Because this reaction corresponds to uncompleted oxidation of UQH_2_ in the forward direction of the operation of the Q_o_ site, it is referred to as a *semiforward* reaction.

The second scenario assumes the sequence of reactions schematically shown in Figure 6B. The UQH_2_ molecule at the Q_o_ site undergoes oxidation and two electrons are transferred to the 2Fe2S cluster and heme *b*_L_, leading to UQ bound at the Q_o_ site. Since the electron transfer from heme *b*_L_ to heme *b*_H_ is not possible, the electron from heme *b*_L_ is transferred back to the UQ molecule forming SQ_o_. At the same time the reduced 2Fe2S cluster is still at the position close to the Q_o_ site and is able to interact with SQ_o_. As a result the SQ_o_-2Fe2S state is generated. Because this reaction corresponds to uncompleted reduction of UQH_2_ in the reverse direction of the operation of the Q_o_ site, it is referred to as a *semireverse* reaction.

The role of heme *b*_L_ in the formation of SQ_o_-2Fe2S is different for both the mechanisms. The *semiforward* reaction occurs when heme *b*_L_ is unable to accept the electron from SQ_o_; thus, the redox-active heme *b*_L_ is not required for the formation of the SQ_o_-2Fe2S state. Conversely, the *semireverse* reaction obligatorily requires presence of the redox-active heme *b*_L_ to enable back electron transfer to the Q_o_ site to form the SQ_o_-2Fe2S.

In view of these considerations, H198N provided an attractive opportunity to test the contribution of the *semireverse* reaction to the formation of the SQ_o_-2Fe2S state. The high-spin, high-potential heme *b*_L_ in this mutant, once accepting the electron, will preferably stay reduced, and thus is unable to pass electron further to heme *b*_H_ at a significant rate. This means that its redox activity required for the catalytic turnover is lost. At the same time, this heme is unable to transfer electron back to the Q_o_ site. It follows that the H198N mutant provides conditions that should favor the formation of the SQ_o_-2Fe2S if the *semiforward* reaction is a dominant mechanism, but should discourage the formation of SQ_o_-2Fe2S if the *semireverse* reaction is dominant. Thus, if the SQ_o_-2Fe2S state was detected in the H198N mutant of Cyt*bc*_1_, then both *semiforward* and *semireverse* would be potentially responsible for the formation of this state. This was clearly not the case. The observation that the H198N mutation leads to complete disappearance of the SQ_o_-2Fe2S signal under the tested conditions implies that the *semireverse* reaction can be considered the dominant mechanism for the formation of this state in WT Cyt*bc*_1_. This mechanism involves electron transfer from heme *b*_L_ back to the ubiquinone bound to the Q_o_ site followed by its interaction with the reduced 2Fe2S cluster.

We note that this scenario prevails even if heme *b*_L_ was able to pass electron to heme *b*_H_ at a significant rate in H198N, which, given that antimycin was present at the Q_i_ site, would eventually lead to a reduction of both hemes *b*_L_ and *b*_H_. In this case, as long as heme *b*_L_ was unable to transfer electron back to the Q_o_ site, the SQ_o_-2Fe2S signal would still be absent if occurrence of the *semireverse* reaction was required to generate it.

We also note that our conclusion requires an assumption that proton paths and proton transfers are not different in the H198N mutant and WT Cyt*bc*_1_. In addition, one should bear in mind that there is still a possibility that the SQ_o_-2Fe2S state can be generated in the H198N mutant but it was not detected in our experiments. This would mean that either the efficiency of its formation, i.e., contribution of the *semiforward* mechanism is much lower than the contribution of the *semireverse* mechanism, or the lifetime of the SQ_o_-2Fe2S state under conditions of altered ligand of heme *b*_L_ is much shorter than the lifetime of this state in WT. If so, then the decay of the SQ_o_-2Fe2S state must have taken place within the deadtime of the method (i.e., before a sample became frozen, see the Materials and Methods). If this alternative explanation was true, it would immediately suggest a change in a mechanism of SQ_o_-2Fe2S formation when properties of heme *b*_L_ were altered in the mutant. In other words, the presence of oxidized heme *b*_L_ in antimycin-inhibited Cyt*bc*_1_ from *R. capsulatus* would greatly stabilize the SQ_o_-2Fe2S state, whereas the reduced or disabled heme *b*_L_ would eliminate or greatly destabilize this state.

## Summary and Conclusion

In this work we constructed a mutant H198N that targeted one of the His ligands of heme *b*_L_. We found that this mutation leads to the conversion of HALS heme *b*_L_ into the high-spin and high-potential form, which was indicated from the analysis of optical and EPR spectra of isolated enzymes. While H198N binds properly the substrates at the Q_o_ site, the specific redox properties of heme *b*_L_ in this mutant made the heme unable to transfer electrons to heme *b*_H_ on a catalytically relevant timescale. Consequently, the enzymatic activity of Cyt*bc*_1_ in H198N was lost. We then used this mutant to test the formation of the SQ_o_-2Fe2S state under nonequilibrium conditions (light-activated chromatophores) when heme *b*_L_ cannot perform normal electron-relay function between the Q_o_ site and heme *b*H.

Careful analysis of various chromatophore samples established that the SQ_o_-2Fe2S signal can be efficiently generated in illuminated chromatophores containing antimycin-inhibited WT Cyt*bc*_1_, provided that the samples are optically transparent and cytochrome *c*_2_ molecules were not lost during the preparation of the chromatophores. Monitoring of the apparent shift in the midpoint potential of the UQ/UQH_2_ couple induced by light activation of RC provides convenient means for verifying the effectiveness of setting the nonequilibrium conditions in the analyzed samples, independently of the analysis of the SQ_o_-2Fe2S signal. In H198N chromatophores, for which this method turned out to be unsuitable, imposing the nonequilibrium conditions was found to result in a large, negative shift of the apparent *E*_m_ of the 2Fe2S cluster (a similar shift in the apparent redox midpoint potential of the 2Fe2S cluster was observed in WT Cyt*bc*_1_ inhibited by myxothiazol). This provided an alternative method for verifying the effectiveness of light activation of RC and additionally confirmed their efficient coupling with Cyt*bc*_1_ *via* cytochrome *c*_2_ resulting in the oxidation of 2Fe2S.

Comparative redox titrations of light-induced WT and H198N chromatophores revealed that the H198N mutant failed to form detectable amounts of the SQ_o_-2Fe2S state under the same conditions at which WT Cyt*bc*_1_ generated a large amount of SQ_o_-2Fe2S. This failure implicates that the *semireverse* mechanism that leads to the SQ_o_-2Fe2S state is the dominant over the *semiforward* reaction in WT Cyt*bc*_1_ (Sarewicz et al., 2010). According to the *semireverse* mechanism, the electron transfer from the low-spin heme *b*_L_ to the UQ bound at the Q_o_ site (*semireverse* reaction) results in the formation of the semiquinone that is spin-coupled to the reduced 2Fe2S, forming the SQ_o_-2Fe2S state detectable by EPR.

## Data Availability

The raw data supporting the conclusion of this article will be made available by the authors, without undue reservation.

## Abbreviations

Cyt*bc*_1_, cytochrome *bc*_1_; Q, quinone (general); QH_2_, quinol (general); UQ, ubiquinone; UQH_2_, ubihydroquinone; RC, reaction center; 2Fe2S, [2Fe-2S] Rieske iron–sulfur cluster; SQ_o_, semiquinone at the Q_o_ site; SQ_o_-2Fe2S, semiquinone spin-coupled to the reduced 2Fe2S.
