# Supplementary material for: The High-Spin Heme b L Mutant Exposes Dominant Reaction Leading to the Formation of the Semiquinone Spin-Coupled to the [2Fe-2S]+ Cluster at the Qo Site of Rhodobacter capsulatus Cytochrome bc 1
Source: Front Chem. 2021 May 7;9:658877. doi: 10.3389/fchem.2021.658877 (PMC8138165; doi:10.3389/fchem.2021.658877)
Supplement: Supplementary file 1 [file DataSheet1.PDF]

Supporting information

to

The high-spin heme b_L mutant exposes dominant reaction leading to formation of the semiquinone spin coupled to $[2Fe\text{-}2S]^+$ cluster at the Q_o site of *Rhodobacter capsulatus* cytochrome bc_1

Effect of loss of cytochrome c_2 from chromatophores on generation of the SQ_o-2Fe2S state

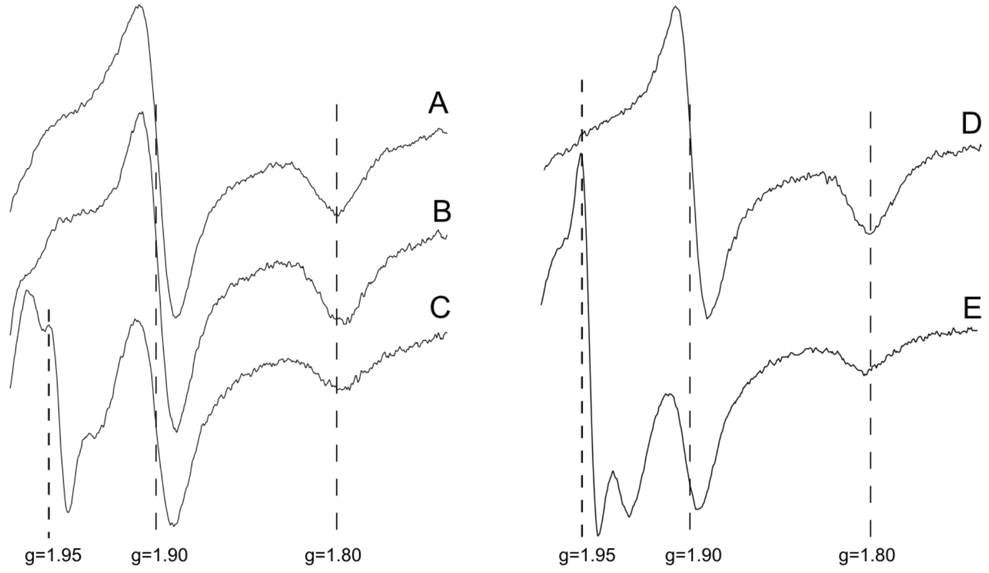

Fig. S1 Effect of loss of cytochrome c_2 from chromatophores obtained from bacteria cells centrifuged with excessive RCF. The EPR spectra were registered for redox poised chromatophores at pH 8 and E_h in which the SQ_o-2Fe2S signal is expected to be the highest. A) The spectrum of chromatophores in prepared in dark. B) A spectrum of the chromatophores prepared in light. C) The spectrum of the chromatophores sonicated after addition of horse cytochrome *c,* prepared in light. For comparison the spectra of WT chromatophores isolated in optimal conditions in dark and light are shown on right (D and E, respectively).

Checking the purity of the isolated WT and H198N mutant of Cytbc_1.

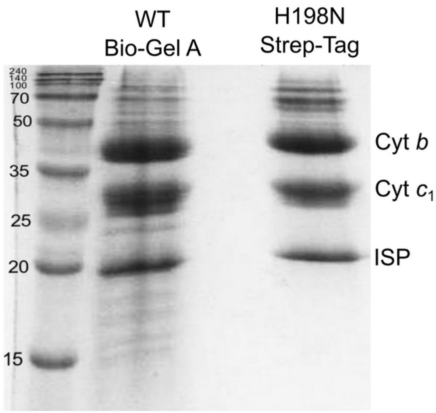

Fig. S2. SDS page analysis of the isolated H198N mutant using Strep-Tag affinity column *(right)*. For comparison the WT protein isolated using ion-exchange chromatography is shown on the left. The first lane on left shows a set of mass standards.

Comparison of heme b_L redox potentials in WT and H198N mutant through analysis of EPR properties of the SQ_i signal.

In one of our recent works we showed that a semiquinone at the Q_i site (SQ_i) of Cytbc_1 generated by addition of the reduced quinone analog decybenzohydroquinone (DBH_2) is detected by EPR spectroscopy as two forms that differ in the spin-lattice relaxation rates [1]. This difference is a result of dipolar interaction of SQ_i with a fast relaxing heme b_H and can be exposed by the use of different microwave power (MP) in CW EPR measurement of the SQ_i signals (fig. S3and S4). In WT Cytbc_1 both, slow- and fast-relaxing forms are present, indicating an electron equilibration between two hemes b_H in the dimeric Cytbc_1 (fig. S3 black) see ref. [S1]. It implies that upon formation of sub-stoichiometric SQ_i at the Q_i sites of Cytbc_1 one of two hemes b_H in the dimer can be reduced, either, adjacent to SQ_i or that on the neighboring monomer. In the later case the oxidized (thus paramagnetic) heme b_H is adjacient to SQ_i which due to dipolar interaction with the heme exhibits a relatively fast relaxation. Therefore, a part of the SQ_i population is fast-relaxing and while the rest of SQ_i species remains slow-relaxing. At the same time hemes b_L in WT Cytbc_1 remain oxidized due to the lower redox potential than heme b_H. However, hemes b_L must still have the redox potential which allows creation of a bridge for electron transfer between the monomers to allow equilibration between hemes b_H [2,3].

Introducing the H198N mutation to Cytbc_1 may lead to following scenarios: i) formation of the heme knock-out, which does incorporate heme b_L to the Cytbc_1 structure and thus a bridge for electron transfer between the monomers does not exists or ii) the heme is incorporated but its redox properties and/or spin state is significantly different than those in WT Cytbc_1, thus perturbing normal electron flow through the hemes *b* in Cytbc_1. If the fist scenario is true then the whole population of SQ_i should be slow-relaxing, and if the second scenario is true then expected results will be more complex. As shown in figure S4, it is clear that in the H198N mutant the whole population of SQ_i is fast relaxing, since the radical signal cannot be saturated at any available MP. This immediately implies that the H198N mutation increases the redox potential of heme b_L to a significantly higher values than heme b_H. This in turn retards normal electron transfer from heme b_L to heme b_H required for efficient catalytic activity. Additionally, as discussed in the main text, it appears that the H198N mutation changes the spin-state of heme b_L, which in the oxidized state appears as an axial high-spin (H-S) heme. The characteristic g = 5.96 transition assigned to this H-S heme is not detected in WT (fig. S5 upper blue spectrum), while it is clearly detected in air-oxidized samples of H198N mutant (fig. S5 bottom blue spectrum). Interestingly, this transition disappears completely after addition of DBH_2 to the sample (fig. S5 bottom black spectrum), which implies that the H-S heme becomes reduced under conditions of SQ_i formation.

This conclusion gains additional support from measurements of the EPR signals of hemes *b* in WT and H198N mutant after addition of DBH_2 to the samples (fig. S5). In WT, hemes b_L remain fully oxidized upon reduction with DBH_2, while hemes b_H are partially reduced (fig. S5, upper spectra). Under the same conditions, in H198N mutant, the g = 5.96 signal completely disappears after reduction with DBH_2, while hemes b_H remain fully oxidized.

Summarizing all the EPR measurements one may conclude that H198N mutation:
a) converts heme b_L from HALS to an axial H-S form;
b) increases the redox potential of H-S heme to a higher values than heme b_H;
c) the increase of the redox potential and/or spin state retards electron transfer from H-S heme to heme b_H, precluding normal UQH_2 oxidation by this mutant;

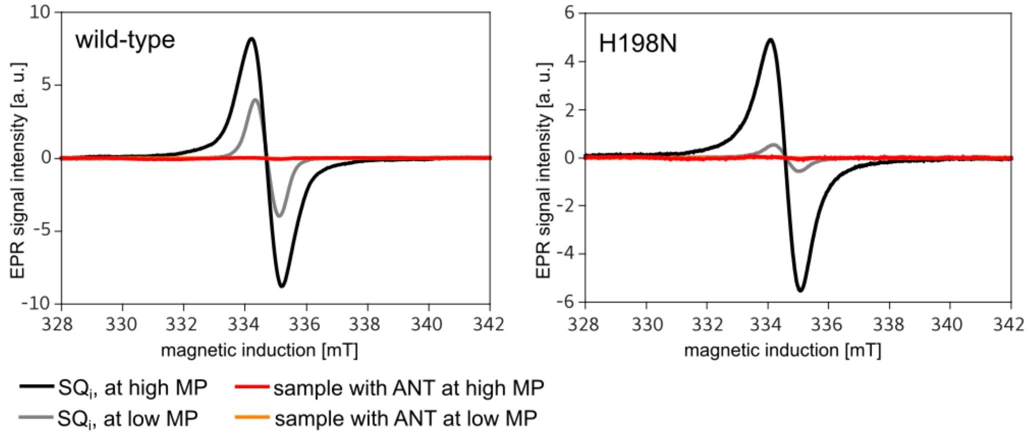

Figure S3. Semiquinone radical signal at the Q_i site of WT cytochrome *bc*$_1$ (*left*) and the c*ytb*:H198N mutant (*right*). The signals were measured at high (*black*) and low MP (*gray*). Addition of antimycin completely abolished the SQ_i signal irrespective of the used MP, which proves the Q_i-origin of these transitions.

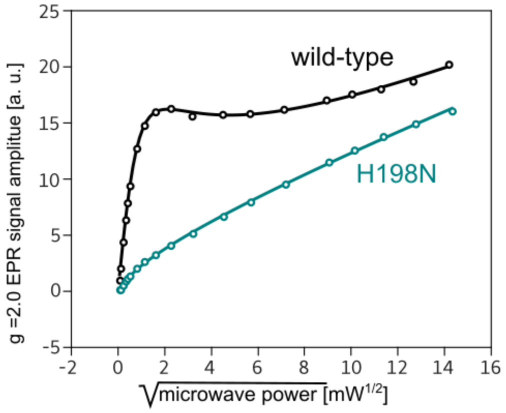

Figure S4. Comparison of MP saturation curves of the semiquinone at the Q_i site for WT (*black*) and the H198N mutant (turquoise).

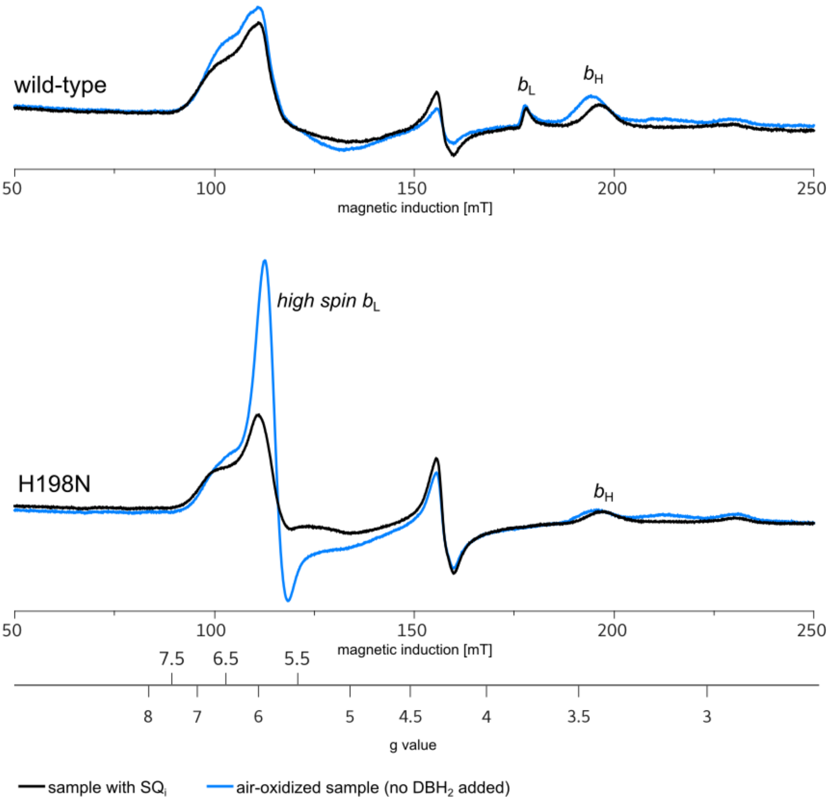

Figure S5. EPR transitions of high- and low-potential hemes in WT (*upper*) and H198N mutant (*bottom*) of Cyt*bc*$_1$. Spectra were measured for air-oxidized (*blue*) samples and in the presence of DBH_2 which generates SQ_i.

Materials and methods (supplementary)

High and low microwave power X-band spectra of SQ_i were measured with 65 and 0.13 mW microwave power and frequency 9.4 GHz, respectively. Modulation amplitude 6 G was used in all measurements of the SQ species. The temperature dependence of the SQ_i amplitude in H198N was measured with microwave power of 65 mW. The microwave power saturation profiles of SQ_i were fitted using formulas described in the literature [4], data were fitted assuming a contribution from one saturable and one unsaturable components as described in [1].

The metal centers were measured using the following parameters: temperature 10 K, microwave power 2 mW, frequency 9.4 GHz and modulation amplitude of 6 G.

Preparation of EPR samples and EPR measurements of isolated cytochrome *bc*$_1$.

Generation of SQ_i in isolated cytochrome bc$_1$.

SQ_i in cytochrome *bc*$_1$ variants was generated by addition of decylubiquinol (DBH_2, a water-soluble analog of QH_2) to the sample of isolated enzyme with the Q_o site inactivated by myxothiazol,

according to the protocol described in [1]. The final concentrations of Cyt*bc*$_1$, myxothiazol and DBH_2 in the reaction mixture were 70, 300 and 420 μM, respectively. Samples for negative control were prepared with 400 μM antimycin in the reaction mixtures. Before injection of DBH_2, these samples were incubated for 20 min on ice after addition of antimycin.

References

[1] S. Pintscher, R. Pietras, M. Sarewicz, A. Osyczka, Electron sweep across four b-hemes of cytochrome *bc*$_1$ revealed by unusual paramagnetic properties of the Q_i semiquinone intermediate, Biochim. Biophys. Acta - Bioenerg., 1859 (2018) 459–469.

[2] M. Świerczek, E. Cieluch, M. Sarewicz, A. Borek, C.C. Moser, P.L. Dutton, A. Osyczka, An electronic bus bar lies in the core of cytochrome *bc*$_1$, Science, 329 (2010) 451–454.

[3] M. Czapla, E. Cieluch, A. Borek, M. Sarewicz, A. Osyczka, Catalytically-relevant electron transfer between two hemes b_L in the hybrid cytochrome *bc*$_1$-like complex containing a fusion of *Rhodobacter sphaeroides* and *capsulatus* cytochromes *b*, Biochim. Biophys. Acta, 1827 (2013) 751–760.

[4] C. Altenbach, W. Froncisz, R. Hemker, H. Mchaourab, W.L. Hubbell, Accessibility of nitroxide side chains: absolute Heisenberg exchange rates from power saturation EPR, Biophys. J., 89 (2005) 2103–2112.
